# Supplementary material for: Effects of aging and risk-taking behaviors on fatal injuries among old motorcyclists in Taiwan: Evidence from 2011 to 2022
Source: PLoS One. 2025 Sep 8;20(9):e0328832. doi: 10.1371/journal.pone.0328832 (PMC12416733; doi:10.1371/journal.pone.0328832)
Supplement: Attachment — Submitted filename: 3-17 response letter.pdf [file pone.0328832.s001.pdf]

Dear Editors,

We sincerely appreciate the valuable comments and suggestions provided by the editors on our previous submission (PONE-D-25-10055). Please find our detailed responses below. We would be grateful for the opportunity to have our revised manuscript reviewed again.

Best regards,

Chih-Wei Pai (Prof)

Graduate Institute of Injury Prevention and Control College of Public Health

Taipei Medical University

Editor's comments: We recognize that you obtained ethical approval from your local ethics committee after you began the study: your ethics approval document is dated 04 March 2024, while personal data were collected and accessed beginning on 11/11/2023. For human subjects research, PLOS ONE requires that authors obtain ethics committee approval before beginning the study, and we do not accept retrospective ethical approval.

Author's response: Thank you for your feedback, we appreciate the editor's comments and suggestions. We sincerely apologize for the typing error in our previous manuscript. The correct statement should be that personal data were collected and accessed beginning on 11/11/2024, not 11/11/2023. This aligns with our ethical approval, which was granted on 04 March 2024, before the study commenced. We have attached both a clean version of the manuscript and a version with track changes for review. This correction has been incorporated into our current submission. We appreciate the opportunity to clarify this point and will make the necessary correction in the manuscript.
